# Clinical, Functional, and Midterm Survival Analysis on Sigma Curved Plus Ultracongruent Polyethylene Insert in Primary Total Knee Arthroplasty: A Retrospective Study

**DOI:** 10.7759/cureus.11519

**Published:** 2020-11-17

**Authors:** Neelam Reddy, Mukesh K Saini, Gattu Naresh, Ajay Thakur, Rajesh Podili, Jayavardhan Reddy

**Affiliations:** 1 Orthopaedics (Arthroplasty), Star Hospitals Hyderabad, Hyderabad, IND; 2 Orthopaedics, Star Hospitals Hyderabad, Hyderabad, IND

**Keywords:** knee arthroplasty, ultracongruent insert, anterior stabilized knee, deep dished polyethylene, knee prosthesis survival

## Abstract

Background: Posterior-stabilized (PS) total knee arthroplasty (TKA) poses problems such as the need for intercondylar notch bone resection as well as cam and post wear and patella clunk. Owing to its heightened anterior profile, an ultracongruent polyethylene insert prevents the excessive posterior translation of tibia in the case of a deficient or scarified posterior cruciate ligament (PCL). This study aimed to determine whether an ultracongruent insert provides satisfactory clinical and functional outcomes and midterm survival benefits.

Methods: Based on the reviewed medical records of 200 patients, 240 primary TKA cases involving the use of Sigma Curved Plus (DePuy International, Ltd., Leeds, UK) ultracongruent insert were retrospectively enrolled in this study. Follow-up data were used to evaluate the clinical and radiological outcomes and to conduct a Kaplan-Meier survival analysis.

Results: The mean follow-up duration for 224 knees was 5.8 years (range 5-6.5 years). A revision was made due to infection in two patients and due to periprosthetic fractures in two other patients. The mean knee flexion improved from 101.97° ± 9.43° (range 85°-125°) to 125.75° ± 9.58° (range 100°-140°) at the final follow-up. The mean Knee Society score improved from 43.1 ± 9.76 to 88.3 ± 3.2, and the function score improved from 44.95 ± 7.26 to 90.16 ± 3.71. None of the patients showed radiographic loosening of either insert component, but 22 (5%) patients showed radiolucent lines (<2 mm). The Kaplan-Meier analysis showed that the five-year survival of the insert with an endpoint of revision for any reason was 98.1% (confidence interval, CI, 95.7-99.6%).

Conclusion: The Sigma Curved Plus insert showed a low failure rate with good clinical, functional, and midterm survival outcomes in comparison to standard outcomes reported in earlier studies. Further follow-up studies are warranted to determine whether the insert’s performance is maintained in the long term.

## Introduction

Total knee arthroplasty (TKA) has been a reliable treatment for severe knee arthritis. The best knee replacement is one with a kinematics that is as close as possible to that of a normal knee. Among the several factors affecting knee kinematics, apart from variations in surface geometry and the posterior cruciate ligament (PCL), ligament balance in the sagittal plane and the coronal plane is of utmost importance for long-term survival and kinematics. However, the evidence on how best to deal with the PCL at the time of knee replacement surgery is equivocal as balancing the PCL is sometimes difficult, and the technique employed to deal with PCL varies from surgeon to surgeon [1].

The PCL is the primary restraint to the posterior translation of the tibia, and it acts as the primary stabilizer of the knee. Retaining a functional PCL offers the following potential advantages: it allows for femoral rollback, it improves stair climbing ability and proprioceptive function, it reduces patellar complications, antero-posterior shear forces, and bone component interface stresses [2,3]. Nevertheless, retention is not always an option, and substitution of the PCL may be required in cases where it is found to be too attenuated to be functional or too tight causing accelerated polyethylene wear due to high contact stresses. The potential advantages of sacrificing the PCL include ease of joint balancing and avoidance of future instability with PCL rupture after a cruciate-retaining (CR) TKA [2]. PCL substitution in primary TKA has traditionally relied on cam and post (cam-post) mechanisms in the form of a posterior-stabilized (PS) design. The advantages of PCL substitution are increased range of motion (ROM), less premature wear of the polyethylene component, and better knee kinematics.

The notable disadvantages of PCL-substituting systems are the additional bone resection from the intercondylar notch of the femur, potential wear at the cam-post interface, patella clunk syndrome, potential midflexion instability, and abnormal gait patterns. However, short- and long-term outcome analysis showed that the clinical outcomes of PS and CR knees did not significantly differ [2,3].

The Sigma Curved Plus insert, which became commercially available in 2004, is intended to provide a surgeon with another option regarding the management of PCL intraoperatively, that is, either to retain or sacrifice the PCL. The curved plus insert does not have a post unlike a stabilized insert, but it functions similar to a PS design owing to its heightened anterior profile, which is designed to prevent the excessive posterior translation of tibia in case of a deficient or scarified PCL [2,4]. Given its advantages, namely, intraoperative flexibility, subluxation resistance, bone preservation, minimal synovial irritation, post wear prevention, and reduced patellar clunk, this design has recently gained attention from most surgeons. Hence, we used the curved plus insert when PCL was incompetent or PCL needed sacrifice for knee balance, and we assessed the clinical, functional, and midterm survival outcomes of the implant with this insert.

## Materials and methods

An institutional review board waived the approval in view of retrospective observational study involving data collection from medical records without changing routine treatment care protocols for all patients who underwent a TKA with Sigma Curved Plus insert performed by the senior author between January and December in 2013. Medical records were reviewed to collect the demographic, pre-operative clinical and radiological, and intra- and post-operative information of the patients. A total of 200 patients (240 knees) met the inclusion criteria and included in this study.

Inclusion criteria were (i) patients who underwent TKA for advanced osteoarthritis/rheumatoid arthritis of the knee; (ii) patients aged 50-80 years; (iii) patients implanted with press-fit condylar (PFC) Sigma knee prosthesis coupled with Curved Plus ultracongruent polyethylene insert. Exclusion criteria include (i) patients with post-traumatic arthritis; (ii) patients whose knee has an extra-articular deformity, malignancy, and hemoglobinopathies; (iii) patients aged below 50 years and above 80 years.

Implant

**Figure 1:**
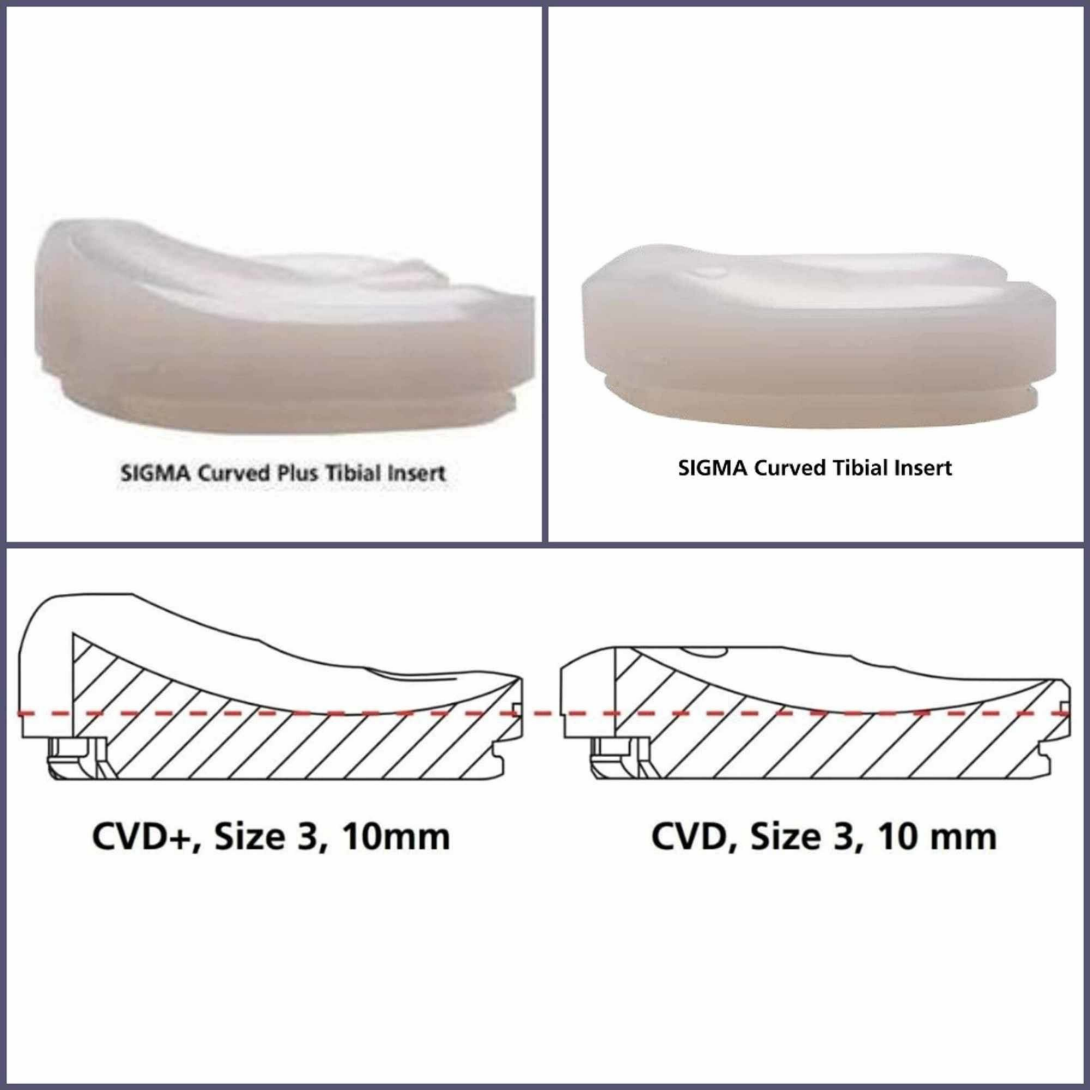
The ultracongruent polyethylene insert Sigma Curved Plus polyethylene insert (left). The anterior profile is heightened compared with the CR insert curved (right) to achieve increased subluxation resistance. CR: cruciate retaining.

The PFC Sigma knee system (DePuy Orthopaedics, Warsaw, Ind) is a second-generation continuation of the PFC knee (Johnson & Johnson, New Brunswick, USA). Sigma is a modular prosthesis with a cobalt-chromium femoral component articulating against a polyethylene insert mounted on a titanium tibial tray with a cruciform stem. The Sigma Curved Plus insert is designed to be used by surgeons who need to sacrifice the PCL for balancing, want to save the bone of the femur, and not to substitute the PCL with a traditional cam-post mechanism as in PS knee [5]. Surgeons may also use this insert in cases where PCL is deficient or compromised. To achieve greater anterior subluxation resistance, the anterior lip height of the Curved Plus insert was increased by an average of 3 mm relative to that of the traditional CR insert, which is also known as Sigma curved (Figure 1). Moreover, the dwell point was moved posteriorly in order to increase the amount of force and the distance required before a potential subluxation can occur. To increase the conformity with the CR femoral components, the radius of curvature on the articulating surface of the insert was decreased. The increased anterior lip height and the decreased radius of the curvature provide the necessary constraint and stability during a cruciate-sacrificing procedure without the use of a post-cam mechanism.

Surgical technique

**Figure 2:**
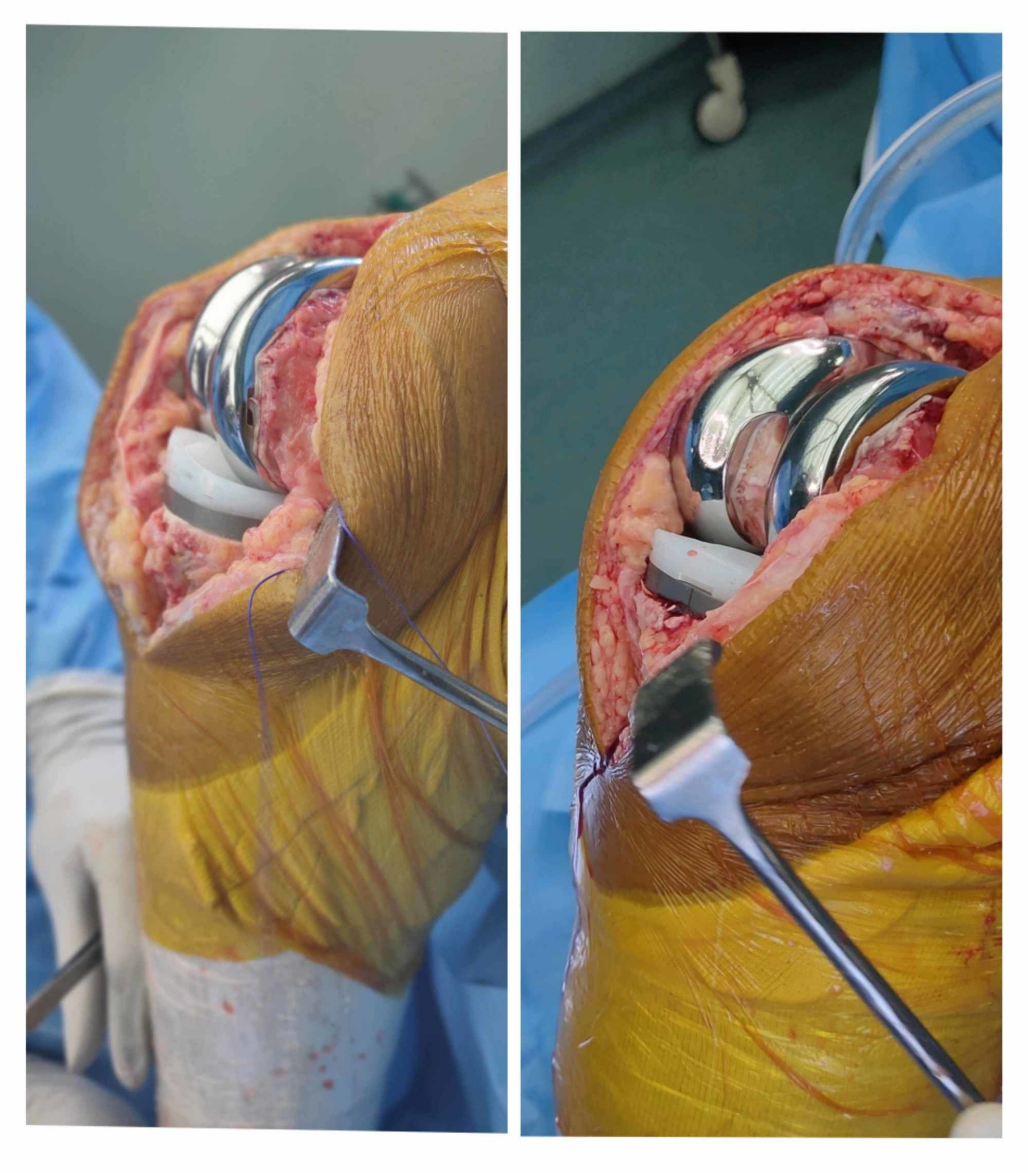
Intraoperative photographs Intraoperative images showing the ultracongruent insert (left) and the usual CR insert (right). CR: cruciate retaining.

Surgery was performed under spinal anesthesia in unilateral TKA and under combined spinal and epidural anesthesia in bilateral TKA. An inflatable tourniquet with a pressure set at 100 mm Hg above the systolic blood pressure was used routinely. The standard operative techniques were used, including the anterior midline skin incision with a medial parapatellar approach; removal of hypertrophic soft tissue, menisci, and the osteophyte; and resection of the anterior cruciate ligament. The distal femur bones were first cut by nearly 9 mm according to the intramedullary alignment system, with 5° valgus in varus deformity and 3° valgus in valgus deformity. The femur size was measured with a cutting block, and an appropriate anterior, posterior, and chamfers cut was made. Then, the tibia bones were cut perpendicular to the long axis of the tibia with a 3°-5° posterior slope through the extramedullary alignment system; the flexion and extension gaps were measured with a distracter; when necessary, the medial or the lateral structures were released so as to obtain a balanced knee movement during flexion and extension. The patella was resurfaced in patients who had severe cartilage damage; the rest of the patients underwent osteophyte removal and denervation. Once the trial implants were placed, the PCL was evaluated for both competence and balance. With the knee set at 90° of flexion using the pull-out and lift-off test, the PCL was found intact and balanced through a negative posterior drawer test and based on the femur - insert contact at half of the joint translation. If these conditions were not satisfied, the PCL was considered either incompetent or excessively tight. When the PCL was considered incompetent or overly tight (requiring subsequent PCL recession or release), an anterior-stabilized Curved Plus insert with 9-11 mm anterior lip (size-dependent) was chosen as the bearing surface (Figure 2). Patellar tracking was subsequently assessed. All prostheses were implanted using hand-mixed antibiotic cement. The wound closed in layers after achieving homeostasis. The tourniquet was then released. All patients received antibiotic prophylaxis shortly before inflation of the tourniquet; also, they received pre- and post-operative thrombo-embolism prophylaxis using low-molecular-weight heparin, aspirin, and full-length graded compression elastic stockings. Drains were not used routinely.

From the first postoperative day, all patients started doing active knee motion exercises and began standing at the bedside or walking with crutches or with a walker twice daily for 30 min each time under the supervision of a physical therapist. The patients used crutches or walkers with full weight-bearing for six weeks and thereafter used a cane as needed.

Clinical evaluation

Clinical evaluations were performed preoperatively and at one, three, and six months postoperatively and then annually. None of the patients reported any wound healing or patellar tendon-related problem. The mean follow-up duration was 5.8 years (range 5-6.5 years). All clinical data at the time of each follow-up were recorded. We obtained the Knee Society clinical and function scores for each knee. Active knee motion, both with the patient in the supine position and during weight-bearing, was determined with the use of a standard (60 cm) clinical goniometry before the operation and at the time of the review. A simple subjective pain grading was used to assess the anterior knee pain (grade 0 - no pain; grade 1 - mild pain; grade 2 - moderate pain; grade 3 -severe pain).

Radiographic evaluation

Antero-posterior radiographs (with the patient standing and lying supine), lateral radiographs, and skyline patellar radiographs were obtained preoperatively and at each follow-up visit. These radiographs were assessed for the alignment of the limb (tibio-femoral angle), the position of its components, the posterior slope of the tibia component, the level of the joint line, and the presence and location of radiolucent lines at the bone-cement interface according to the recommendations of the Knee Society [6]. The prosthesis-bone interface was evaluated for the presence and/or progression of radiolucent lines.

The progression of a radiolucent line was defined as the increase in length and/or width by 2 mm or greater in sequential radiographs. Component alignment and position were compared with previous radiographs to determine whether subsidence or loosening occurred. Joint spaces were evaluated for asymmetry or changes in thickness (as measured with a magnifying ruler), which can indicate polyethylene insert wear.

Statistical analysis

Survivorship assessment was performed using the Kaplan-Meier analysis. A paired t-test or Wilcoxon signed-rank test was used for data comparison depending on whether the data had a normal distribution. The level of significance was set at P < 0.05. Survivorship analysis was performed to determine the cumulative rate of survival of the implant during the study period, and the results were reported with a 95% confidence interval (CI) level. The endpoint for the analysis was the revision surgery for any reason.

## Results

Patients demographics

Out of the 200 patients, 130 were female and 70 were male. A total of 160 patients underwent unilateral TKA, and 40 patients underwent bilateral TKA. The mean age at surgery was 61.58 years (50-80 years), and the indications for surgery were primary osteoarthritis (201 knees, 83.7%) and rheumatoid arthritis (39 knees, 16.3%). Out of 160 unilateral TKA, 141 were done for OA and 19 for RA. Likewise, out of 40 bilateral surgeries (i.e., total 80 TKA), 30 were done for OA and the rest 10 were done for RA as an indication.

Of the 240 knees, 40 had incompetent PCL before surgery, 120 were subjected to intraoperative complete release, and 80 were subjected to partial release. Twenty-four knees (10%) had patellar resurfacing, and the rest underwent osteophyte removal and denervation. The mean tourniquet time was 50±15 min.

Survival analysis

The mean follow-up duration in our study was 5.8 years (range 5-6.5 years). Eight patients expired within five years from the date of surgery. Three patients expired due to chronic renal failure, and two others expired because of myocardial infarction. One patient died due to periampullary carcinoma three years after the knee surgery. Additionally, one patient died due to a road accident and another patient died due to intracranial bleeding. All of the patients who expired received a unilateral knee replacement, leaving 232 knees available for the final follow-up. Of these knees, eight were lost to follow-up (address untraceable). One knee underwent a two-stage revision because of a late infection with methicillin-resistant *Staphylococcus aureus* 27 months after surgery. In another knee, a single-stage revision was performed at 31 months due to a deep infection. In yet another knee, a periprosthetic fracture of the femur occurred 17 months after the operation, and this fracture was treated successfully with revision TKA as both components were loose. In another case, a periprosthetic fracture with severe bone loss required hinge replacement. Both patients who had fractures are walking well now (Table 1).

**Table 1:** Patient characteristics and outcomes of the patients requiring revision

S. No	Age	Sex	Cause for revision	Timing of revision	Outcome
1	52	M	Periprosthetic fracture	18 months	Good
2	63	F	Infection	27 months	Good
3	72	M	Infection	31 months	Good
4	57	M	Periprosthetic fracture	45 months	Good

The Kaplan-Meier survivorship analysis results showed that the Sigma Curved Plus knee prosthesis demonstrated a 98.1% (CI 95.7-99.6%) survivorship with failure for any reason at a minimum five-year follow-up (Table 2). The patients who died, the patients lost to follow-up, and the failed cases were included in the survival analysis so that the follow-up includes all the outcomes accounted for in the calculation. Given that no aseptic loosening or mechanical failure was noted, the survival analysis involving these conditions as endpoints will yield a 100% survivorship for the Sigma Curved Plus knee implant.

**Table 2:** Five-year survivorship analysis of PFC Sigma Curved plus knee prosthesis The number of knees enrolled at the beginning of the study = 240. Kaplan-Meir analysis was done accounting for failures and lost to follow-up cases. There were eight deaths*, eight lost to follow-up* and four failures^#^ which were all unilateral cases. CI: confidence interval, PFC: press-fit condylar.

Time period (year)	At risk	Unavailable (lost to follow-up/died)*	Revised/ failed^#^	Survival probability estimate	95% CI	
					Lower limit	Upper limit
First	240	3	0	1.000	0.971	0.999
Second	237	5	1	0.995	0.964	0.998
Third	231	4	1	0.990	0.964	0.998
Four	226	2	2	0.981	0.964	0.998
Fifth	222	2	0	0.981	0.957	0.996

Clinical and functional outcome

For the remaining knees (224), the mean knee flexion improved from 101.97° ± 9.43° (range 85°-125°) preoperatively to 125.75° ± 9.58° (range 100°-140°) at the final follow-up (P < 0.0001). Of these knees, 15.4% had a ROM of 100°-120°, 64% had a ROM of 120°-130°, and 20.6% had a knee ROM of more than 130°. Preoperatively, a flexion contracture of 13.08° ± 7.42° (range 0°-30°) was noted, and it improved at the final follow-up to a mean of 2.44° ± 1.68° (range 0°-5°; P-value 0.0016 for Wilcoxon signed-rank test). A total of 171 knees had grade 0 anterior knee pain, 42 had grade 1 anterior knee pain, and 11 knees had grade 2 anterior knee pain. No knee had grade 3 anterior knee pain, and no crepitus was palpable in any of the patients. No patients demanded revision or treatment for anterior knee pain

The mean Knee Society score improved from 43.1 ± 9.76 to 88.3 ± 3.2, and the Knee Society function score improved from 44.95 ± 7.26to 90.16 ± 3 (P < 0.001).

Radiological outcome

The radiographs of the 224 knees were available for analysis at a five-year follow-up. The mean preoperative tibio-femoral alignment was 13.66° ± 10.28° varus (range 30° varus to 20° valgus), and the postoperative alignment was 3.97° ± 2.42° valgus (range 0°-7° valgus) as calculated by drawing the anatomical axis of the femur and tibia on scannogram films. Based on the definition of radiological loosening as the radiolucency (>2 mm) in all zones of a component or the progression of lucent lines from earlier films, no components were found to be radiographically loose. In the radiographs, the radiolucent lines (<2 mm) were seen under the tibia tray in 21 cases. However, these lines were non-progressive in sequential radiographs, and no alteration in the coronal and sagittal plane was noted or no revision was required due to aseptic loosening of the prosthesis.

## Discussion

Clinical and radiological outcomes and survivorship are the two most important measures that establish the usefulness of implants in arthroplasty. The current series, given the considerably low number of patients lost to follow-up (<4%), allows for a fair evaluation of the efficacy of the design features incorporated into the investigated implant system.

Osteolysis and radiolucencies are additional concerns affecting the longevity of TKA. Bozic et al. found that 21% of their patients had 1-mm radiolucencies at five- to eight-year follow-up of a modern knee design [7]. By contrast, Bertin found that 8.6% of their patients had a non-progressive radiolucency at a five- to seven-year follow-up of a similar modern knee design [8]. In the present study, we found that 9% of the patients had non-progressive osteolysis (radiolucency <2 mm), and we could not find distinguishing radiographic characteristics in terms of alignment; moreover, their Knee Society scores were no different from those of the entire group. However, on average, these patients were younger (55 years) based on the average age of the entire group. Younger age has been associated with increased incidences of osteolysis. However, a longer follow-up of this series is needed to ascertain the patients’ role in aseptic loosening.

Polyethylene wear and the resulting osteolysis play an important role in the early failure of a knee implant. The survivorship of Sigma Curved Plus knee was 98.1%, indicating a low polyethylene wear rate and can be taken as a surrogate to the quality of the polyethylene. Furthermore, there were no cases of aseptic loosening, again indicating fewer amounts of wear debris and osteolysis in the mid-term.

High-flexion implants have consistently demonstrated an average knee flexion greater than 120° (Table 3), although the clinical advantage of an increased flexion is debatable [9-16]. In the present study, the mean knee flexion was 125°; this indicates the outcome is comparable to the high flexion design with this insert. This flexion is also comparable to that of other ultracongruent inserts. Song et al. compared an ultracongruent insert with a conventional CR knee prosthesis; the ultracongruent insert demonstrated a 130.8° flexion, which is higher than the 128.7° flexion in the CR group [17].

**Table 3:** Literature review of the range of knee flexion for high-flexion knee prosthesis PS: posterior-stabilized, CR: cruciate-retaining, PFC: press-fit condylar, RPF: rotating platform flexion.

S. No.	Author	Year of publication	Total knee implant	Mean knee flexion angle (degrees)
1	Kim et al. [9]	2005	High flex PS	138.6
2	Nutton et al. [10]	2008	High flex PS	127
3	Seon et al. [11]	2009	High flex PS	135.3
4	Endres and Wilke [12]	2010	High flex CR	122
5	Kim et al. [13]	2012	High flex CR	139
6	Maniar and Singh [14]	2012	PS-RPF	130
7	Lee et al. [15]	2013	High flex PS	132.2
8	Sancheti et al. [16]	2016	INDUS	128.17
9	Present study	2019	PFC-Sigma	125

Survivorship of various implants at a five-year midterm follow-up ranged from 90% to nearly 100% [18-24]. In their study of 9200 TKA, Rand and Ilstrup suggested that the probability of survival of any implant is approximately 97% both at 5 and 10 years, whereas a recent meta-analysis reported a pooled survivorship of 98.4% at a five-year follow-up [24,25]. These reported survivorship rates are comparable to the survivorship of the Sigma Curved Plus knee (98.1%; Table 4). Good survivorship and low revision rates are a function of surgical technique as well as implant design. Deep flexion activities are common in our country, and although our patients were advised to avoid such activities, there are situations wherein they do flex their knees beyond 100°. Although we cannot always force patients to follow our advice, Sigma Curved Knee does make these deep flexion activities safer due to its design stability compared to CR implants in a well-balanced knee, and this design stability is probably one of the most important factors leading to its high survivorship despite deep flexion. No cam-post impingements further reduced the total wear rates, leading to the extended longevity of the implant.

**Table 4:** Comparison of survivorship of TKA implants PFC: press-fit condylar, PS: posterior-stabilized, RPF: rotating platform flexion, TKA: total knee arthroplasty.

Serial No	Authors	Year of publication	Implant	Number of years	Survivorship (%)
1	Front-Rodriguez et al. [18]	1997	Total condylar series	21	90.77
2	Shen et al. [19]	2009	PS - all polyethylene PFC Sigma	5.9	93.55
3	Asif and Choon [20]	2006	Cemented PFC	6	94.0
4	Kim et al. [21]	2007	Fixed bearing-cruciate retaining	5	94.0
5	Meftah et al. [22]	2012	RPF	5	97.7
6	Hopley and Dalury [23]	2014	Pooled survivors of sigma Knee	5	98.5
7	Sancheti et al. [16]	2016	INDUS knee	5	98.6
8	Present Study	2019	PFC Sigma Curved Plus	5	98.1

The complications in our series were also comparable with those in other implants wherein the infection rate was 1% (2 out of 224) [23,24]. No case of early loosening or implant subsidence was noted in the present cases. However, the incidence of periprosthetic fractures was lower in our series than in other series [23,24] likely because our patients remained less aggressive in terms of their activities even after a successful surgery. Moreover, radiolucent lines were seen under the tibia tray, but these lines were non-progressive similar to previous results.

The incidence of patella-femoral pain was 23.66% (53/224), 79.24% of which was mild pain. By contrast, some authors reported an incidence of knee pain ranging from 8% to 50% [25,26]. Systematic reviews have reported similar rates of anterior knee pain in both resurfaced and non-resurfaced groups [27,28]. Some studies have associated the anterior knee pain with the severity of cartilage damage, whereas others have attributed it to dysfunctional muscular coordination in thigh muscles [26,29]. In our series, we performed selective resurfacing of patella depending on the severity of cartilage damage, and this approach is probably the reason behind the observed lower rates of severe anterior knee pain.

Our study has a few limitations. No comparison group was included; however, this study was designed purely to conduct a survival analysis of the implant. Few patients were lost to follow-up, but these patients were considered when calculating the survivorship and were accounted for in our analysis. Our sample was comparatively smaller as this investigation was a single-center study. However, as in the early days of the implant, maximum cases were done at one center, and so proper follow-up could be conducted at that center. Another limitation is the relatively short-term follow-up of the implant.

## Conclusions

Based on the clinical and functional results achieved in our cases at the end of five years and based on the 98.1% survivorship of the implant, it can be concluded that TKA involving the use of ultracongruent polyethylene insert (Sigma Curved Plus) demonstrated excellent midterm analysis results. These results are comparable with those obtained for similar implants; however, a long-term follow-up (10-15 years) involving multi-center trials with a focus on aseptic loosening and functional outcomes are necessary to determine the long-term efficiency of this implant in terms of stability, functional outcome, and survival.
